# Evaluation of Polygenic Risk Scores for Prediction of Prostate Cancer in Korean Men

**DOI:** 10.3389/fonc.2020.583625

**Published:** 2020-10-22

**Authors:** Jong Jin Oh, Eunae Kim, Eunjin Woo, Sang Hun Song, Jung Kwon Kim, Hakmin Lee, Sangchul Lee, Sung Kyu Hong, Seok-Soo Byun

**Affiliations:** ^1^Department of Urology, Seoul National University Bundang Hospital, Seongnam, South Korea; ^2^Department of Urology, Seoul National University College of Medicine, Seoul, South Korea; ^3^Procagen, Seongnam, South Korea

**Keywords:** prediction, Genomics, polygenic risk score, Koreans, prostate cancer

## Abstract

**Aims:**

The purpose of this study is to evaluate an aggregate influence of prostate cancer (PCa) susceptibility variants on the development of PCa in Korean men by using the polygenic risk score (PRS) approach.

**Methods:**

An analysis of 1,001 cases of PCa and 2,641 controls was performed to: (i) identify potential PCa-related risk loci in Koreans and (ii) validate the cumulative association between these loci and PCa using the PRS. Subgroup analyses based on risk stratification were conducted to better characterize the potential correlation to key PCa-related clinical outcomes (e.g., Gleason score, prostate-specific antigen levels). The results were replicated using 514 cases of PCa and 548 controls from an independent cohort.

**Results:**

Genome-wide association analysis from our discovery cohort revealed 11 candidate single-nucleotide polymorphisms (SNPs) associated with PCa showing statistical significance of *p* < 5.0 × 10^–5^. Seven variants were located at *8q24.21* (rs1016343, rs16901979, and rs13252298 in *PRNCR1*; rs4242384, rs7837688, and rs1447295 in *CASC8*; and rs1512268 in *NKX3*). Two variants located within *HNF1B* (rs7501939 and rs4430796) had a significant negative association with PCa risk [odds ratio (OR) = 0.717 and 0.747, *p* = 6.42 × 10^–7^ and 3.67 × 10^–6^, respectively]. Of the six independent SNPs that remained after linkage disequilibrium (LD) pruning, the top four SNPs best predicted PCa risk with an area under the receiver operating characteristic curve (AUC) of 0.637 (95% CI: 0.582–0.692). Those with top 25% polygenic risk had a 4.2-fold increased risk of developing PCa compared with those with low risk.

**Conclusion:**

Eleven PCa risk variants in Korean men were identified; PRSs of a subset of these variants could help predict PCa susceptibility.

## Introduction

Prostate cancer (PCa) is the second most common cancer in men worldwide (1). In South Korea, the number of men diagnosed with PCa increased rapidly between 1999 and 2009, with an annual increase of 12.9% (2). Between 2009 and 2017, the incidence of PCa grew by 0.8% per year, resulting in the fourth highest incidence and the third highest prevalence of cancer types in South Korean men. Interestingly, while the incidence of PCa continues to increase in South Korea, it has gradually declined for other common cancers (e.g., stomach, lung, colon, liver, and thyroid) (2). Importantly, despite having a lower incidence compared with Western populations, the proportion of advanced-stage PCa in Koreans and other Asian populations is higher (3). Given the associated public health burden caused by PCa, there is a growing need to identify high-risk groups to generate effective screening and prevention strategies for PCa.

Genetic profiling can be a useful clinical instrument to help determine an individual’s risk for PCa. Multiple large-scale genome-wide association studies (GWASs) have led to the identification of more than 170 single-nucleotide polymorphisms (SNPs) underlying susceptibility to PCa (4). Multiple PCa-associated SNPs have been estimated to explain 33% of the risk of developing PCa (5). SNPs associated with PCa can vary greatly by population; several attempts have focused on identifying SNPs that may be associated with PCa in Asian populations. One large-scale meta-analysis reported Asian-specific PCa-associated SNPs from two Asian (Japanese and Chinese) populations (6). Additionally, a Korean population-based exome-wide study identified five significant SNPs across four distinct loci (7). Each of the common SNPs identified by the GWAS confers small-to-modest effects on the development of PCa (8).

An aggregate influence of SNPs can be assessed by generating a polygenic risk score (PRS)—a measure of the cumulative contribution of individual SNPs carried by a particular person (9). Even if individual variants have only small effect sizes, their cumulative impact on risk of PCa can be significant, thus making the PRS a potentially powerful tool for the prediction of PCa (9, 10). Eeles et al. (11) generated a PRS using 68 established PCa risk variants and reported that men in the top 1% of the risk distribution had more than a four-fold increased risk for PCa compared with those in the average risk distribution range. Another PRS study demonstrated that men in the top 10% of the risk distribution had a 3.19-fold higher risk of PCa compared with those with average risk (12). In this study, we assessed the cumulative impact of PCa-related genetic variants in predicting the risk of PCa using weighted PRS in the Korean male population.

## Materials and Methods

### Ethics Statement

After approval by our institutional review board (B-1312/232-302), all analyses were performed following the Declaration of Helsinki. All study participants provided written informed consent.

### Study Population and Genome-Wide Association Study Genotyping

For the discovery of PCa-associated candidate SNPs, we initially obtained genotype data of 1,001 PCa samples from a single tertiary hospital and 2,210 controls from the Korean Association Resource (KARE) study as part of the Korean Genome and Epidemiology Study (KoGES). Gleason score (GS) was identified with ≥12 core transrectal ultrasound prostate biopsy and/or radical prostatectomy (RP) specimens; specimens were reviewed by a single experienced uro-pathologist. Controls were: (i) men who had never been diagnosed with PCa, (ii) residing in the cities of Ansung or Ansan, (iii) recruited between 2001 and 2002, and (iv) 60 years of age or older. More detailed information about the cohort is available in a previously published article (13).

Genotyping of PCa samples was performed with the HumanExome BeadChip 12v1-1 system (Illumina, Inc., San Diego, CA, United States) (14, 15). Details about SNP content and selection strategies can be found at http://genome.sph.umich.edu/wiki/Exome_Chip_Design. Genotype calling was performed using Illumina’s GenTrain version 2.0 clustering algorithm with the GenomeStudio software (V2011.1). Cluster boundaries were determined using Illumina’s standard cluster file. To improve the accuracy of variant calling, manual reclustering and visual inspection were conducted for genotypes based on the CHARGE clustering method (14). Sample quality control was carried out to exclude samples with genotyping rates < 95%, heterozygosity, and cryptic relatedness. Markers were excluded based on the following criteria: (i) monomorphic in our samples, (ii) with missing call rate > 5%, (iii) with minor allele frequency (MAF) < 5%, or (iv) significantly deviated from the Hardy–Weinberg equilibrium (*p* < 1.0 × 10^–6^) using PLINK 1.9. After quality control, 24,023 variants from 984 PCa cases and 2,194 control subjects remained for subsequent analysis. To evaluate allelic associations with PCa development, logistic regression analysis was performed given case/control status after adjusting for age as a covariate using the PLINK software.

For the evaluation of polygenic risk in an independent cohort, 516 cases of PCa and 546 controls from Chungbuk National University Hospital were initially obtained. Candidate PCa-associated SNPs showing suggestive significance were included for analyses. The genotyping of these SNPs was performed using the Fluidigm 192.24 Dynamic Array TM IFC and Biomark HD systems. Duplicates and negative controls were included in each 96-well plate for quality control. The average concordance rate between the duplicate samples was >99%. After sample quality control and exclusion of control samples <60 years of age, we included 514 cases and 124 controls for further analysis.

For PRS construction, the most significant SNP in each linkage disequilibrium (LD) block was further selected *via* LD clumping (*R^2^* < 0.001). The PRS was calculated by summing effect size-weighted counts of risk alleles for PCa-associated SNPs. An individual *i*’s PRS is defined as follows:

$$S_{i}=\frac{\sum_{\left(j=1\right)}^{M}X_{i\,j}\,{\hat{\beta }}_{j}}{n},$$

where *X*_*j*_ is the number of risk alleles (0, 1, or 2) for the variant *j*, β_*j*_, is weighting [log(OR)] of the variant *j*, which is obtained from the discovery set, and *n* is the total number of the variants included. For those SNPs whose minor alleles showed protective effects on PCa, we converted their minor alleles to major alleles as risk alleles, which results in positive weight values for all variants. As an increasing number of top SNPs were included (*n* = 3–6), predictive abilities of their PRS were compared using the area under the receiver operating characteristic (ROC) curve (AUC) (16). Improvement in AUC between ROC curves were tested using Delong’s method (17). Youden’s Index (*J*), which is defined as (sensitivity + specificity-1), was acquired to capture predictive performance and also to determine the cutoff PRS at the maximum *J* (18). The analyses were performed using the R package “*pROC*.”

## Results

For cases in the discovery set, the median age was 68 years and the median prostate-specific antigen (PSA) level was 9.19 ng/ml. Most of the cases (90%) were diagnosed with PCa with a GS of 7 or higher. The controls were 3 years younger (median age of 65 years) compared to the case group (*p* < 0.0001; Table 1). The average body mass index (BMI) was significantly higher in cases compared to controls by 1.1 kg/m^2^ (*p* < 0.0001). For the validation set, the median ages of cases and controls are 69 and 67 years (*p* = 0.04). Eighty percent of the cases in the validation set are diagnosed with PCa with a GS ≥ 7.

**TABLE 1:** Baseline characteristics of the study population.

**Characteristics**	**Discovery set (*n* = 3,178)**			**Validation set (*n* = 630)**		
	**Prostate cancer (*n* = 984)**	**Control (*n* = 2,194)**	***p*-value**	**Prostate cancer (*n* = 506)**	**Control (*n* = 124)**	***p*-value**
Median age (years) ± SD	68 ± 7.22	65 ± 3.63	<0.0001	69 ± 7.46	67 ± 5.39	0.04
Median PSA levels (ng/mL) ± SD	9.19 ± 138.89	N.A.	–	9.11 ± 501.28	N.A.	–
Mean BMI (kg/m) ± SD	24.49 ± 8.26	23.39 ± 3.38	<0.0001	N.A.	N.A.	–
**Gleason score (n,%)**						
6	102 (10.4)	–		96 (19)	–	
7	699 (71.4)	–		252 (49.9)	–	
8	77 (7.9)	–		73 (14.5)	–	
9	94 (9.6)	–		78 (15.4)	–	
10	7 (0.7)	–		6 (1.2)	–	

*SD: standard deviation; PSA: prostate-specific antigen.*

Genome-wide association analysis from the discovery cohort revealed 11 SNPs associated with PCa, showing genome-wide suggestive significance of *p* < 5.0 × 10^–5^ (Table 2). Seven variants were located at *8q24.21* (rs1016343, rs16901979, and rs13252298 in *PRNCR1*; rs4242384, rs7837688, and rs1447295 in *CASC8*; rs1512268 in *NKX3*). The most significantly associated SNP was rs1016343 [odds ratio (OR) = 1.598 (1.424–1.793); *p* = 1.46 × 10^–15^] located within the *PRNCR1* locus. Two variants located within *HNF1B* (rs7501939 and rs4430796) had a significant negative association with PCa risk (OR = 0.717 and 0.746, *p* = 6.42 × 10^–7^, and 3.67 × 10^–6^, respectively). Of the top 11 SNPs, six variants were positively associated with PCa risk (OR > 1), while five showed negative associations (OR < 1). Of the three variants in *PRNCR1*, one variant (rs13252298) showed protective effects compared to two other susceptibility variants (rs1016343 and rs16901979).

**TABLE 2:** PCa-associated SNPs (genome-wide significance <5.0 × 10^–5^) identified by GWAS (*n* = 10).

**SNP**	**CHR**	**BP**	**Minor allele**	**Gene**	**OR [95% CI]**	***p*-value**
rs1016343	8	128093297	A	*PRNCR1*	1.598 [1.424–1.793]	1.46 × 10^–15^
rs16901979	8	128124916	A	*PRNCR1*	1.512 [1.336–1.71]	5.09 × 10^–11^
rs4242384	8	128518554	C	*CASC8*;*LOC105375754*	1.559 [1.359–1.787]	1.97 × 10^–10^
rs7837688	8	128539360	A	*CASC8*;*LOC105375754*	1.581 [1.37–1.823]	3.42 × 10^–10^
rs13252298	8	128095156	G	*PRNCR1*	0.701 [0.617–0.796]	4.58 × 10^–8^
rs1447295	8	128485038	A	*CASC8*;*LOC105375754*	1.463 [1.275–1.678]	5.76 × 10^–8^
rs7501939	17	36101156	A	*HNF1B*	0.717 [0.629–0.817]	6.42 × 10^–7^
rs4430796	17	36098040	G	*HNF1B*	0.746 [0.659–0.844]	3.67 × 10^–6^
rs1512268	8	23526463	A	*NKX3.1*;*LOC107986930*	1.303 [1.161–1.462]	6.51 × 10^–6^
rs2735839	19	51364623	A	*KLK2*;*KLK3*	0.766 [0.682–0.86]	6.84 × 10^–6^
rs2016588	6	159425707	A	*LOC105378083*	1.268 [1.138–1.414]	1.69 × 10^–5^

**FIGURE 1:**
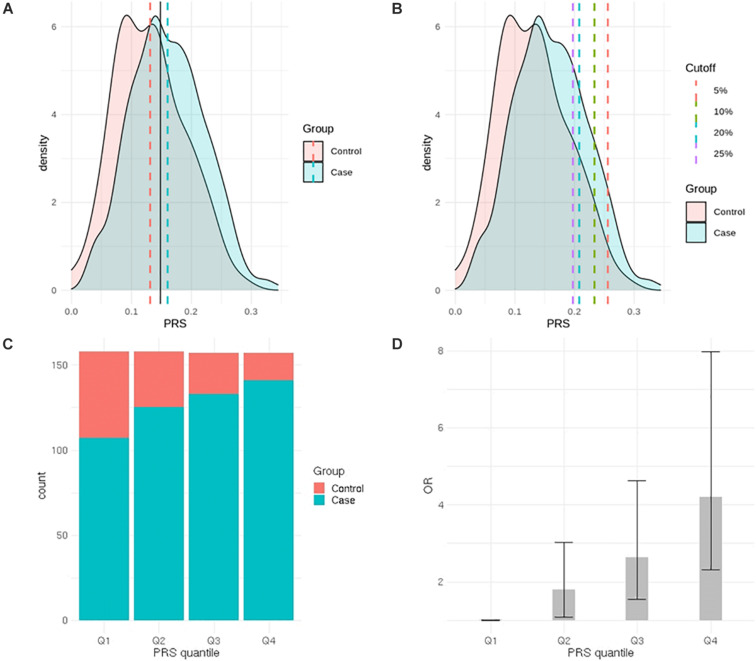
Comparison of prostate cancer (PCa) polygenic risk between PCa cases and controls. **(A)** Distribution of the polygenic risk score (PRS) in PCa cases and controls (black solid line represents cutoff value at the maximum J). **(B)** Distribution of the PRS in PCa cases and controls (each dashed line represents high-risk PRS group—top 2.5, 5, 10, and 20%). **(C)** Distribution of cases and controls according to PRS quantiles. **(D)** Odds ratio for developing PCa according to PRS quantiles.

**FIGURE 2:**
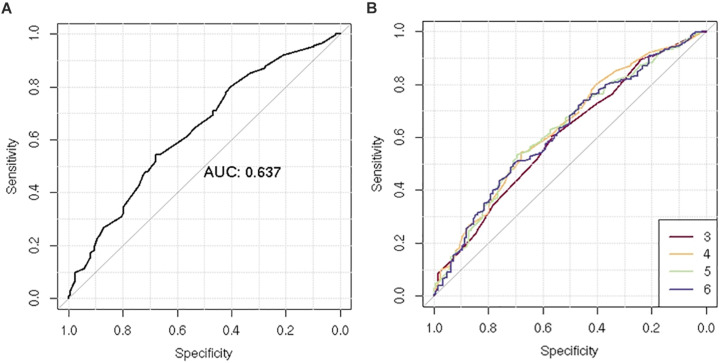
Receiver operating characteristic (ROC) curve of the polygenic risk score (PRS) for the prediction of prostate cancer (PCa). **(A)** Comparison of ROC curves according to increasing number of single-nucleotide polymorphisms (SNPs) included for PRS calculation (*n* = 3–6). **(B)** ROC curves of the PRS using four SNPs.

After LD clumping of the top 11 SNPs, the remaining six variants (Table 3) were included for PRS calculation. The mean PRS was significantly higher in PCa cases compared to controls across all SNP sets (Table 4 and Figure 1A). Polygenic risk of PCa was best predicted with an AUC of 0.637 (95% CI: 0.582–0.692) when calculated upon the top four independent SNPs (Figure 2A). The PRS model built with four SNPs showed significant improvement in AUC compared to the model with three SNPs according to DeLong’s tests (*p* = 0.005; Table 4 and Figure 2B). However, it was not significantly different from the AUCs of PRS models computed upon five or six SNPs. Meanwhile, the set of the top four SNPs yielded the second highest maximum Youden’s index (*J* = 0.221), with sensitivity and specificity of 0.543 and 0.677, respectively, which was slightly lower than that of the top five SNPs (*J* = 0.227; Table 4). Overall, the specificity of the PRS at the maximum Youden’s index was higher compared to its sensitivity, with the exception of the model built with three SNPs (sensitivity: 0.607, specificity: 0.565).

**TABLE 3:** Candidate PCa-associated SNPs retained after LD clumping for construction of PRS (*n* = 6).

**SNP**	**CHR**	**BP**	**Gene**	**P**	**SNPs in LD**	**Risk allele**	**Minor allele = risk allele**	**Weight [log(OR)]**
rs1016343	8	128103937	*PRNCR1*	1.46 × 10^–15^	rs13252298, rs16901979	A	Y	0.469
rs4242384	8	128518554	*CASC8*;*LOC105375754*	1.97 × 10^–10^	rs1447295, rs4242382, rs7837688	C	Y	0.444
rs7501939	17	36101156	*HNF1B*	6.42 × 10^–7^	rs4430796	C	N	0.333
rs1512268	8	23526463	*NKX3.1*;*LOC107986930*	6.51 × 10^–6^	–	A	Y	0.265
rs2735839	19	51364623	*KLK2*;*KLK3*	6.84 × 10^–6^	–	G	N	0.266
rs2016588	6	159425707	*LOC105378083*	1.69 × 10^–5^	–	A	Y	0.237

**TABLE 4:** Comparison of the predictive performance of PRS according to different SNP sets.

**Top N SNPs included for PRS calculation**	**Mean PRS**		**AUC [95% CI]**	**Maximum Youden’s Index (*J*)**	**Sensitivity**	**Specificity**	Improvement in AUC (*p*)*
	**Case**	**Control**					
4	0.131	0.16	0.637 [0.582–0.692]	0.221	0.543	0.677	Ref
5	0.139	0.163	0.628 [0.574–0.683]	0.227	0.534	0.694	− 0.009 (0.406)
6	0.135	0.155	0.624 [0.569–0.678]	0.208	0.498	0.710	− 0.013 (0.360)
3	0.151	0.181	0.607 [0.552–0.662]	0.171	0.607	0.565	− 0.03 (0.005)

*The ROC curves were compared with the reference ROC curve using DeLong’s test.

When the PRS was computed upon the top four independent SNPs, the upper quantile (top 25%) of the PRS had a 4.2-fold greater risk of developing PCa compared with the reference group (Q1) [OR: 4.2 (2.32–7.98)] (Table 5 and Figure 1C). With a larger number of PCa cases observed in the upper quantiles of the PRS, the Q2–Q4 groups carried significantly higher risk compared to the reference (Q1) group (Table 5 and Figure 1D). High-risk group defined by top 5% to 25% in the distribution had also significantly elevated risk of PCa compared to the remaining population: the top 10% of the PRS had a 3.08-fold risk, and the top 5% had a 3.71-fold risk of developing PCa compared to the remaining population (Table 6 and Figure 1B).

**TABLE 5:** Distribution of cases and controls according to PRS* quintiles.

**Total *N* = 630**	**Q1 (Ref)**	**Q2**	**Q3**	**Q4**
Control, *N* = 124	51	33	24	16
(*n*,%**)	41.13%	26.61%	19.35%	12.90%
Case, *N* = 506	107	125	133	141
(*n*,%)	21.15%	24.7%	26.28%	27.87%
OR [95% CI]	–	1.81 [1.09–3.02]	2.64 [1.54–4.63]	4.20 [2.32–7.98]

*PRS was computed upon four SNPs. **% individuals of each quantile within each cae/control group.

**TABLE 6:** Risk of high PRS groups for development of PCa.

High PRS* group	**Reference group**	**OR [95% CI]**
Top 25%	Remaining 75%	2.61 [1.53–4.72]
Top 20%	Remaining 80%	2.71 [1.50–5.35]
Top 10%	Remaining 90%	3.08 [1.33–8.98]
Top 5%	Remaining 95%	3.71 [1.10–23.14]

*PRS was computed upon four SNPs.

## Discussion

In the development of PCa, genetic susceptibility plays an important role. Stratification of individuals based on their inherited genetic risk can be important in screening and prevention strategies of PCa. The present study used multiple PCa-associated SNPs detected in Korean men to evaluate their predictive ability using the weighted PRS approach.

We identified 11 SNPs associated with PCa showing statistical significance of *p* < 5.0 × 10^–5^. Since the ultimate goal of this study was to evaluate the utility of the PRS as a predictor of PCa risk rather than identifying causal PCa SNPs, we applied a lenient statistical threshold to select candidate SNPs for PRS calculation and thus compared the performance by different PRS models. Those PCa-associated variants included rs1016343, rs16901979, and rs13252298 located at *8q24.21* within *PRNCR1*. The association of rs1016343, which was most significantly associated in this study, has been previously reported in other populations (19, 20); the effect of the variant on PCa in the Korean population (OR = 1.598) were greater compared to that of other populations of European ancestry. Four other variants (rs7837688, rs4242384, rs4242382, and rs1447295) in *CASC8* were also replicated; the effect sizes were comparable to those of other studies. The significant association of the rs1447295 variant has been reported in Japanese and Chinese populations (21, 22). The results presented here are of importance in that they focus specifically on the Korean population.

We identified several variants protective for PCa risk. One variant (rs13252298) located within *PRNCR1* showed protective effects, while the other two *PRNCR1* loci conferred susceptibility to PCa (rs1016343 and rs16901979). It was contrasted with other regions harboring multiple significant SNPs such as *CASC9*, *HNF1B*, and *RFX6*, within which consistent directions of effects were observed. Two variants (rs7501939 and rs4430796) at *17q12* in *HNF1B* showed protective effects on PCa risk, with ORs of 0.717 (0.629–0.817) and 0.746 (0.659–0.844), respectively. Previous studies also reported protective effects for these variants in the European (23) and Korean population (24). However, the associations reported by Gudmundsson et al. (25) were not consistent with this and other studies of European populations, showing susceptibility to PCa with ORs of 1.19 (1.12–1.26) and 1.22 (1.15–1.30) for rs7501939 and rs4430796, respectively. The *HNF1B* gene (formerly known as transcription factor TCF2) is known to, at least in part, regulate the levels of metabolic and hormonal factors in PCa.

Since the previous report on strong cumulative effects of five SNPs on PCa in Korean men (10), many studies have reported significant associations between the PRS and the risk of PCa (19, 26, 27). In the present study, we applied weighted PRS models after excluding controls <60 years of age from the previously available cohort to reduce possible confounding effects due to ages and adjusted for ages as a covariate in analyses. We also compared polygenic risk by different weighted PRS models, which produced an AUC of 0.637 using the top four independent SNPs compared to the AUC of 0.605 using non-weighted PRS using the top five SNPs in the previous study (10). Despite the improvement in predictive performance, our model shows modest performance compared to other large-scale studies such as an international PCa genetics consortium [Prostate Cancer Association Group to Investigate Cancer Associated Alterations in the Genome (PRACTICAL)] that yielded an AUC of 0.67 using 68 genetic variants (5). Still, this study holds significant implications as an efficient tool for screening high-risk group using only a small number of SNPs for PRS calculation: those with the top 25% PRS showed a 4.2-fold increase of developing PCa compared to the low-risk group. It was comparable impact of the PRS built with 68 variants reported by Eeles et al. (11), in which men in the top 1% of the risk distribution had a 4.4-fold increased polygenic risk for PCa compared with those with average risk. Latino men in the top 10% PRS stratum had a 3.19-fold elevated risk compared with those in the average range (12). Despite multiple SNPs commonly found across populations, such discrepancy in genetic risk between populations arises from variations in risk allele frequencies, LD structures, and effect sizes on PCa.

Individually calculated PRSs can facilitate stratification of disease risk, which can be utilized for screening and prevention in clinical practice (8, 9). The conventional PRS approach has been recently extended to polygenic hazard score (PHS), which informs the onset age and age-specific genetic risk of certain diseases (28). Applications of PHS may add valuable information for personalized life planning and disease screening. Furthermore, many previous studies showed that, despite genetics being a non-modifiable risk factor, those at higher polygenic risk have been shown to have reduced risk by lifestyle modifications or clinical interventions in other diseases (29–32). To introduce the PRS into clinical practice, there is a need to improve its predictive value by (i) obtaining more valid summary statistics to be applied for computing the PRS using larger-scale data sets, (ii) applying advanced modeling technologies (such as machine learning and other artificial intelligence methods), (iii) incorporating family history, lifestyle factors, and other clinical factors into modeling, and (iv) including super healthy controls strictly screened for diseases.

This study provides valuable scientific evidence to develop screening and prevention strategies that can identify genetic high-risk groups in Korean men. Nonetheless, we acknowledge that our study had several limitations. First, our sample size is modest compared with other large cohort-based studies, especially those on European populations. Another large-scale study may be necessary for deriving more valid summary statistics used for PRS calculation and further testing its performance. Second, although only a small number of variants were able to screen high-risk PCa groups, the inclusion of even more SNPs may achieve a superior predictive ability. Finally, the validation set is not representative of the Korean male population, as the PCa prevalence is 84% in the validation set, which is considerably higher than that of the Korean male population. Although we defined the lowest PRS quantile group as the reference group to represent the general average-risk population, it could have led to underestimation of polygenic risk in high-risk groups due to the possibly elevated PRS in the reference group. For evaluation of predictive utility and generalizability of our findings, an external validation set representative of the general Korean male population may be warranted.

In conclusion, we identified 11 PCa risk variants in Korean men and report that PRSs using a subset of these variants may be useful for determining an individual’s risk of developing PCa. The addition of individually calculated PRSs effectively increased the accuracy of predicting PCa. Future studies on modifications of polygenic risk by lifestyle factors may add valuable scientific evidence in preventing the development of PCa in which genetics plays a critical role.

## Data Availability Statement

The datasets generated for this study are available upon request to the corresponding author.

## Ethics Statement

The studies involving human participants were reviewed and approved by Seoul National University Bundang Hospital institutional review board. The patients/participants provided their written informed consent to participate in this study.

## Author Contributions

JO, EK, and S-SB conceived, designed, and supervised the experiments. SS, JK, HL, SL, and SH performed the experiments. JO, EK, and EW analyzed the data. JO and EK wrote the manuscript. All authors contributed to the article and approved the submitted version.

## Conflict of Interest

EK and EW was employed by the company Procagen, South Korea. The remaining authors declare that the research was conducted in the absence of any commercial or financial relationships that could be construed as a potential conflict of interest.
